# 

**Published:** 1876-08

**Authors:** 


### CONTENTS:

### ORIGINAL COMMUNICATIONS.

Article I.—Report on Surgery. By Prof. Christopher Johnston, M D.......... 145

Article II.—(Selected.) Demineralized Dentine. By Dr J. E. Cravens.......... 158

Article III—Is the Profession of Dental Surgery holding its own with other Professions and Pursuits? By Dr. Edgar Palmer.............. 165

American Academy of Dental Science—Ninth Annual Meeting. .. 169

North Carolina State Dental Association .................. 170

American Dental Association.......................... 170

American Dental Convention.......................... 170

### SELECTED ARTICLES.

Article IV.—Pressure and Contact as Causes of Dental Decay. By Henry S. Chase, M. D., D. D. S.................................. 171

Article V.—Phases of Professional Development. By W. C. Horne, D. D. S..... 179

Article VI.—The Lead Line of the Gums. By Dr. C. Hilton Fagge............ 183

### EDITORIAL, ETC.

Diseased Germs.............................. 185

The New Metal Gallium.............................. 186

### MONTHLY SUMMARY.

The Cuca or Coca Leaf and its Effects.............................. 187
Boracic Acid for Ringworm.............................. 188
How do Nerves Transmit Impulses.............................. 188
How to Check Coughs.............................. 188
Boracic Acid.............................. 189
Dressing for Burns.............................. 189
Toothache.............................. 189
Proportion of Medical Men to Population in England.............................. 190
Anæsthesia of Drunkenness Recommended.............................. 190
Female Doctors in the National Association.............................. 190
Relation of Alcohal to Animal Heat and Muscular Power.............................. 190
Neuralgia Relieved by Morphia and Atrophia Combined.............................. 191
The Skin and Bodily Temperature.............................. 191
Dangers of Breathing by the Mouth.............................. 192
Medical Diploma for Women.............................. 192
A New Dental Hospital.............................. 192

